# A Spatio-temporal Bayesian model to estimate risk and influencing factors related to tuberculosis in Chongqing, China, 2014–2020

**DOI:** 10.1186/s13690-023-01044-z

**Published:** 2023-03-21

**Authors:** Zhi-Yi Chen, Xin-Yi Deng, Yang Zou, Ying He, Sai-Juan Chen, Qiu-Ting Wang, Dian-Guo Xing, Yan Zhang

**Affiliations:** 1 School of Public Health, Chongqing Medical University, Chongqing, 400016 China; 2 Research Center for Medicine and Social Development, Chongqing Medical University, Chongqing, 400016 China; 3 Innovation Center for Social Risk Governance in Health, Chongqing Medical University, Chongqing, 400016 China; 4 Research Center for Public Health Security, Chongqing Medical University, Chongqing, 400016 China; 5 Office of Health Emergency, Chongqing Municipal Health Commission, No.6, Qilong Road, Yubei District, Chongqing, 401147 China

**Keywords:** Tuberculosis (TB), Bayesian Spatio-temporal model, Temporal trend, Spatial effect

## Abstract

**Background:**

Tuberculosis (TB) is a serious infectious disease that is one of the leading causes of death worldwide. This study aimed to investigate the spatial and temporal distribution patterns and potential influencing factors of TB incidence risk, and to provide a scientific basis for the prevention and control of TB.

**Methods:**

We collected reported cases of TB in 38 districts and counties in Chongqing from 2014 to 2020 and data on environment, population characteristics and economic factors during the same period. By constructing a Bayesian spatio-temporal model, we explored the spatio-temporal distribution pattern of TB incidence risk and potential influencing factors, identified key areas and key populations affected by TB, compared the spatio-temporal distribution characteristics of TB in populations with different characteristics, and explored the differences in the influence of various social and environmental factors.

**Results:**

The high-risk areas for TB incidence in Chongqing from 2014 to 2020 were mainly concentrated in southeastern and northeastern regions of Chongqing, and the overall relative risk (RR) of TB showed a decreasing trend during the study period, while RR of TB in main urban area and southeast of Chongqing showed an increasing trend. The RR of TB was relatively high in the main urban area for the female population and the population aged 0–29 years, and the RR of TB for the population aged 30–44 years in the main urban area and the population aged 60 years or older in southeast of Chongqing had an increasing trend, respectively. For each 1 μg/m^3^ increase in SO_2_ and 1% increase in the number of low-income per 1000 non-agricultural households (LINA per 1000 persons), the RR of TB increased by 0.35% (95% CI: 0.08–0.61%) and 0.07% (95% CI: 0.05–0.10%), respectively. And LINA per 1000 persons had the greatest impact on the female population and the over 60 years old age group. Although each 1% increase in urbanization rate (UR) was associated with 0.15% (95% CI: 0.11–0.17%) reduction in the RR of TB in the whole population, the RR increased by 0.18% (95% CI: 0.16–0.21%) in the female population and 0.37% (95% CI: 0.34–0.45%) in the 0–29 age group.

**Conclusion:**

This study showed that high-risk areas for TB were concentrated in the southeastern and northeastern regions of Chongqing, and that the elderly population was a key population for TB incidence. There were spatial and temporal differences in the incidence of TB in populations with different characteristics, and various socio-environmental factors had different effects on different populations. Local governments should focus on areas and populations at high risk of TB and develop targeted prevention interventions based on the characteristics of different populations.

## Background

Tuberculosis (TB) is an airborne infectious disease caused by organisms of the Mycobacterium TB complex [1]. It is one of the leading causes of death worldwide, posing a major threat to the life and health of all human beings and bringing a high disease burden, especially in less economically developed countries [2]. China, the world’s largest developing country, had the second highest burden of TB in the world, and according to the WHO Global TB Report, the estimated number of new TB cases in China in 2020 was 842,000, accounting for 8.5% of the global total [2]. Although the incidence of TB in China has shown a decreasing trend since 2000 [3], the reported incidence in western China has been among the highest and some areas has shown an upward trend in recent years [4]. Exploring the spatial and temporal characteristics of TB incidence and influencing factors can provide a scientific basis for TB prevention and control.

In recent years, some scholars have explored the spatial and temporal pathogenesis patterns and influencing factors of TB. Studies in Qinghai [5] and Iran [6] explored high-risk aggregation areas for TB incidence using Moran’I statistics, LISA clustering and Spatio-temporal scan statistics, but such methods could not assess the impact of their potential influencing factors on TB incidence. Wang et al. [7] and Mesay et al. [8] used a local geographically weighted regression model (GWR) to analyze the determinants affecting TB in their study, but did not take into account the effect of spatial interactions. The spatial lag model used by Zhang et al. [9] and the spatial error model used by Gehendra et al. [10] in their studies considered the adjacent spatial effects and explored the potential factors of TB on this basis. However, the methods did not take into account the potential role of spatio-temporal interaction factors in the development of TB and could not estimate the relative risk (RR) of TB. The Bayesian spatio-temporal model used in this paper took the spatio-temporal structure as a priori, considered the influence of adjacent temporal and spatial structure, and realized the simultaneous analysis of temporal, spatial, spatio-temporal interactions and potential influencing factors of TB. More uncertainties were taken into account, so that the RR of TB could be estimated more precisely, and the pattern of spatial and temporal variation of its incidence and its influencing factors could be analyzed.

Regarding the influencing factors of TB incidence, some studies have confirmed that poverty and malnutrition were risk factors of TB [2]. However, there was no consensus on aspects such as ecology and some socio-economic factors. Liu et al. [11] found a significant association between SO_2_ and increased risk of TB (RR: 1.046, 95% CI: 1.038–1.054) in their study, whereas a Thai study [12] did not find an association between the two. The study in Beijing found an association between urbanization rate and increased risk of TB (RR: 1.010, 95% CI: 1.003–1.018) [13], while this was not found in a similar study in Hunan Province, China [14]. Differences in study results might be influenced by different population characteristics and geographical settings, and most of the current studies were on the whole population. So, it is necessary to study the spatial and temporal patterns of TB incidence and the influence of potential factors in different subgroups of the population, and explore the possible differences in the influence of various factors on different populations.

Chongqing is located in southwestern China, in the middle and upper reaches of the Yangtze River, with predominantly mountainous terrain, and is a traditional heavy industrial city with the typical characteristics of a dual economic structure of a large city and a large rural area, where the reported incidence of TB is much higher than that of the whole country and has obvious urban-rural regional differences [15]. Therefore, this study collected reported cases of TB in Chongqing from 2014 to 2020, and aimed to investigate the spatial and temporal distribution patterns of TB incidence risk and potential influencing factors by constructing a Bayesian Spatio-temporal model, to identify key areas and key populations affected by TB. And we compared the spatial and temporal distribution characteristics of TB in populations with different characteristics, and to explore the differences in the influence of various social and environmental factors on different populations.

## Methods

### Study setting

**Fig. 1:**
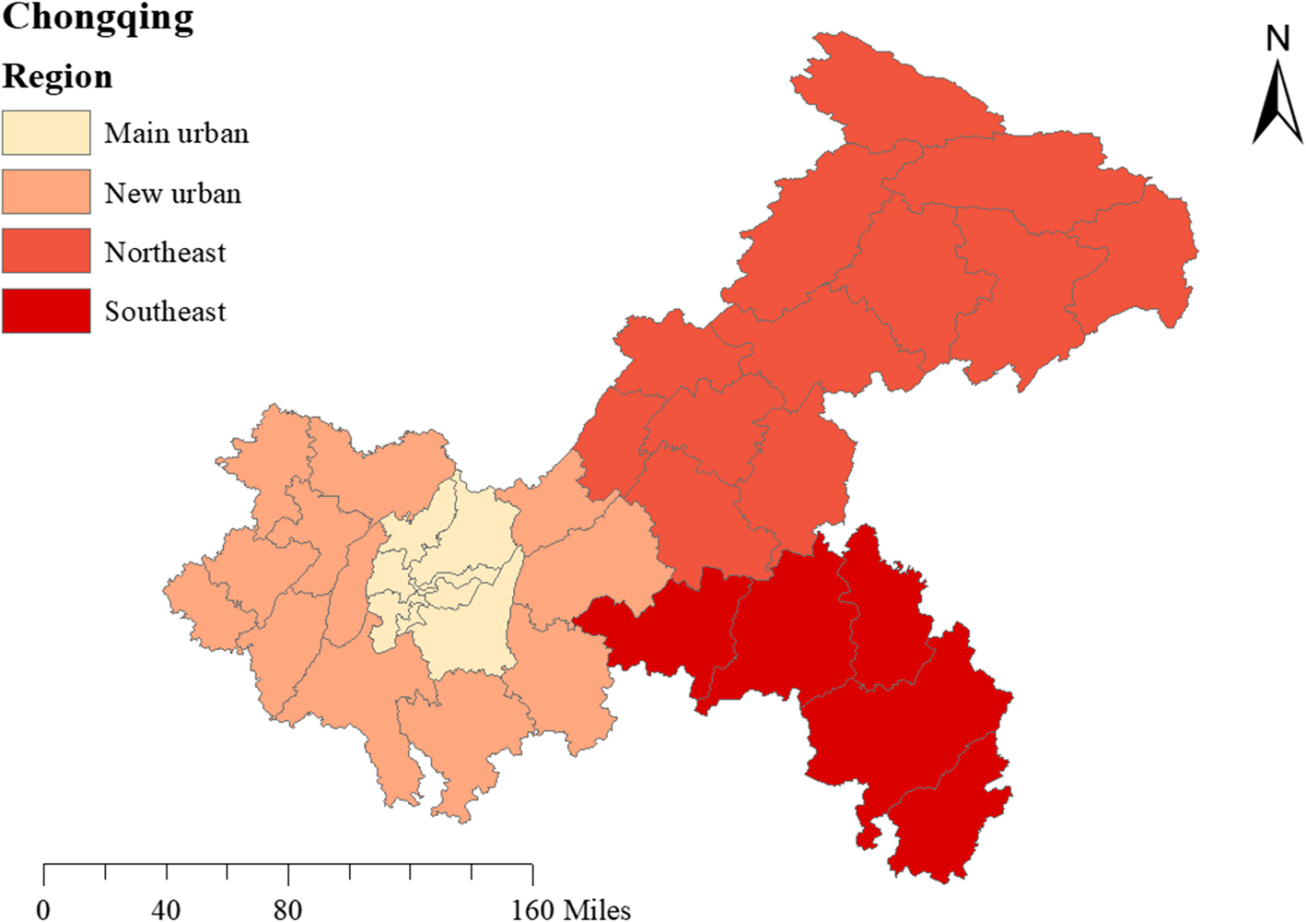
Regional distribution of Chongqing

The study was carried out in Chongqing Municipality, one of the four municipalities directly under the Central Government of China, which is located in the southwest of the Chinese mainland and the upper reaches of the Yangtze River, spanning the transition zone between the Qinghai-Tibet Plateau and the middle and lower reaches of the Yangtze River between 105°11′ ~ 110°11′ east longitude and 28°10′ ~ 32°13′ north latitude. The landform is dominated by hills and mountains, with a large slope area known as a “mountain city”. The terrain of Chongqing is gradually reduced from north and south to the Yangtze River Valley. The northwest and central part are dominated by hills and low mountains, and the northeast is surrounded by Daba Mountain and Wuling Mountain in the southeast. In the study, Chongqing was divided into four districts based on the functional categories, namely main urban area (main urban), new urban area (new urban), northeast Chongqing ecological conservation development zone (northeast), southeast Chongqing ecological protection and development zone (southeast). The specific distribution was shown in Fig. 1.

### Data sources

This study used TB surveillance data in Chongqing from 2014 to 2020, obtained from China Information System for Disease Control and Prevention. The study included all clinically diagnosed and confirmed cases in the case classification [16]. The resident population data of each district and county were from the Statistical Yearbook published by the Chongqing Municipal Bureau of statistics. The four variables of social economy and environment were considered as followings: (1) Environment: including PM_10_(μg/m^3^), SO_2_(μg/m^3^), and NO_2_(μg/m^3^) [11, 17, 18]; (2) Population characteristics: including population density (PD)(people/km^2^), urbanization rate (UR)(%) and proportion of people engaged in agriculture(%)(PEIA); (3) Economic level: including per capita GDP (1000 yuan)(PGDP), disposable income of urban residents (1000 yuan)(DIUR), disposable income of rural residents (1000 yuan)(DIRR) and the number of low-income group per 1000 non-agricultural households (LINA per 1000 persons) [19]. The above data were obtained from the Statistical Yearbook published by the Chongqing Municipal Bureau of statistics and the environmental statistical bulletin issued by the Chongqing Municipal Bureau of ecological environment.

### Statistics analysis

#### Descriptive statistics

In this study, the morbidity of different regions, different gender groups, and different age groups in Chongqing were described and we explored the time series of all TB cases from 2014 to 2020. Then means and standard deviations (SD) were used to describe the impact variables of TB. The impact variables of PD, UR, PGDP, and the LINA per 1000 persons were log-transformed due to the positive skewness distribution of the variables.

#### Spatial-temporal model 

As an initial explanatory analysis, we calculated the global Moran Index and the serial autocorrelation respectively to investigate the spatial and temporal autocorrelation structure of the TB data. After that, we modeled TB cases using a hierarchical Bayesian CAR model analysis based on MCMC simulations and estimated parameters using WinBUGS software. TB cases were treated as count data; thus, assuming that the observed number of cases (*Y*_*ij*_) followed a Poisson distribution [20, 21, 22, 23]. Based on this assumption, the following three models were constructed: Model 1: adding the spatially and non-spatially structured random effect: *θ*_*i*_ =  *exp* (*α*_0_ + *u*_*i*_ + *v*_*i*_). Model 2: adding temporally structured random effect: *θ*_*i*_ =  *exp* (*α*_0_ + *u*_*i*_ + *v*_*i*_ + *g*_*j*_). Model 3: adding spatial-temporal interaction effect: *θ*_*i*_ =  *exp* (*α*_0_ + *u*_*i*_ + *v*_*i*_ + *g*_*j*_ + *psi*_*ij*_). We chose the model with the lowest Deviation Information Criterion (DIC). The final Poisson log-normal model with Spatio-temporal effects and covariates were given by:

$${Y}_{ij}\sim Possion\left({\mu}_{ij}\right)$$

$${\mu}_{ij}={e}_{ij}{\theta}_{ij}$$

$$model3:{\theta}_i=\mathit{\exp}\left({\alpha}_0+{u}_i+{v}_i+{g}_j+{psi}_{ij}\right)$$

$$model4:{\theta}_i=\mathit{\exp}\left({\alpha}_0+{u}_i+{v}_i+{g}_j+{psi}_{ij}+\beta X\right)$$

Here *Y*_*ij*_ represented the number of TB cases and *e*_*ij*_ represented the expected number of TB cases at the *j*_*th*_ time point in the *i*_*th*_ area. *θ*_*ij*_ was the risk of TB at the *j*_*th*_time point in the *i*_*th*_ area. *α*_0_ was an overall intercept term, assigned a flat prior (*α*_0_~*dflat*()). *u*_*i*_ and *v*_*i*_ were spatial structural effects and spatial unstructured effects, respectively. The prior distribution of *u*_*i*_, which denoted the correlation effect between individual districts and counties, was chosen from the conditional autoregressive (CAR) prior with a spatial adjacency matrix W of size N × N proposed by Besag, York, Mollié et al. in 1991 [24]. The conditional distribution of each *u*_*i*_ in this process was a normal distribution with a mean value that was a weighted average of neighboring regions, implying that neighboring districts and counties might have a similar risk of TB. And the diagonal value *w*_*ii*_ = 0,the non-diagonal value *w*_*ij*_ = 1 if region i was adjacent to region j, and the non-diagonal value *w*_*ij*_ = 0 if region i was not adjacent to region j [25]. *v*_*i*_ was the heterogeneity of the region, capturing the part of the overall spatial variability that did not show spatial pattern. And its prior distribution was chosen to be normal (${v}_i\sim N\left(0,{\delta}_v^2\right)$). *g*_*j*_ was the temporal structure effect, capturing the temporal autocorrelation structure, indicating the correlation effect between adjacent years with a prior distribution chosen to be first-order autoregressive (AR(1)). And at this time j time point time effect *g*_*j*_ was only related to the previous moment time effect *g*_*j* − 1_. *psi*_*ij*_ was the Spatio-temporal interaction effect, which referred to the spatio-temporal effect of region i at time j, representing the changes that could not be reflected by spatial and temporal effects alone. It meant the joint effect of spatial and temporal effects on the dependent variable, while both spatial and temporal effects included both structural and non-structural components. The spatio-temporal effects term of the model developed in this study considered the joint effect of temporal structural and spatial unstructured effects on the dependent variable, which meant different regions had different temporal trends and did not have any structure in space [26]. Its structure matrix was *K*_*psi*_ = *K*_*v*_ × *K*_*g*_, and its prior distribution used the second type of prior distribution defined by Knorr-Held [26]. *X* represented the variable factor brought into the model, *β* was its variable coefficient, and its prior distribution was chosen to be non-informative and normal (($\beta \sim N\left(0,{\delta}_{\beta}^2\right)$).

This paper mainly studied the following two aspects:(1) The Spatio-temporal changes of TB relative risk in Chongqing were estimated by using the Bayesian spatio-temporal model (model3) with the lowest DIC. We explored the trend analysis by the temporally random effect (exp(*g*_*j*_)) [27], the RR of spatial effects estimated using spatial effect (exp(*v*_*i*_ + *u*_*i*_)), and the spatial variation trend of TB in 38 districts (counties) of Chongqing by spatial-temporal interaction effect (exp(*psi*)) [28]. Meanwhile, the posterior mean RR of TB of different populations and their time trend from 2014 to 2020 in each district (county) were evaluated. (2) Univariate analysis was used to explore the risk factors for TB by building the models which added the significantly correlated covariates, and the multivariable analysis was conducted to build different population multivariable models in combination with significant variables in univariate analysis (model4).

RR and their 95% credible intervals (95%CI) were computed in WinBUGS14. The Bayesian spatio-temporal model results were presented using ArcGIS’s Getis Ord Gi * statistics.

## Results

### Descriptive statistical analysis

**Fig. 2:**
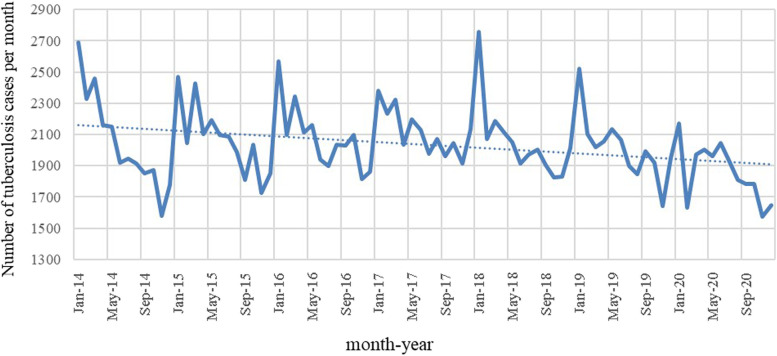
The monthly reported number of TB in Chongqing, China, 2014–2020. The dotted line showed the linear trends of TB over the study period

A total of 170,934 TB cases (confirmed and clinically diagnosed) were reported by the China Information System for Disease Control and Prevention in Chongqing during the study period (2014–2020). The overall temporal trend for TB morbidity was on a decreasing trend, where the maximum occurred in the first year of the study (2014) at 82.81 per 100,000 population, and the minimum occurred in 2020 at 70.19 per 100,000 population. TB cases have shown seasonal variations with the highest number of TB cases reported in January and March in all years (Fig. 2). At the regional level, the highest average annual TB morbidity (156.46/100,000) was in the southeast of Chongqing and the lowest average annual TB morbidity (66.00/100,000) was in the new urban area. At the gender level, the average annual morbidity of males (108.72/100,000) was higher than that of females (47.87/100,000). From an age perspective, the average annual morbidity was the highest in people over 60 years old (116.10/100,000) (Table 1).

**Table 1:** Demographic characteristics of TB (Tuberculosis) morbidity (per 100,000 people) in Chongqing from 2014 to 2020

	2014	2015	2016	2017	2018	2019	2020	Mean
**Area**								
Main urban	69.97	64.5	61.88	67.09	68.78	74.55	64.58	67.34
New urban	75.92	70.54	67.27	63.57	62.28	65.02	57.43	66.00
Northeast	93.67	93.39	105.49	101.34	95.79	76.61	73.73	91.43
Southeast	131.48	135.68	171.64	189.11	171.13	154.69	141.53	156.46
**Gender**								
Male	112.73	109.56	113.47	113.99	109.25	106.45	95.62	108.72
Female	50.62	48.98	48.35	48.88	47.34	47.09	43.81	47.87
**Age(years old)**								
0-	57.48	53.64	53.02	60.29	54.08	50.7	45.11	53.47
30-	76.14	72.44	72.12	65.13	62.95	59.27	52.83	65.84
45-	105.5	102.82	105.48	95.44	94.64	91.55	80.75	96.60
60-	113.07	111.82	119.58	120.47	116.19	118.99	112.61	116.10
Total	82.81	80.07	81.71	82.13	78.99	77.37	70.19	79.04

Table 2 showed the total average concentrations of the pollution factors (PM_10_, SO_2_ and NO_2_) which were 62.03 μg/m^3^, 15.9 μg/m^3^ and 30.47 μg/m^3^, respectively. From the perspective of demographic characteristics, the urban population accounted for nearly two-thirds of the total population, while the agricultural workers accounted for nearly half. The mean value of the economic factors (PGDP, DIUR, DIRR, and LINA) were 57.32(1000 yuan), 31.85(1000 yuan), and 13.71(1000 yuan) and 24.03(per 1000 persons), respectively.

**Table 2:** Descriptive statistics of covariates from 2014 to 2020

Covariate	Mean ± SD	Min	*P*_25_	Median	*P*_75_	Max
**Environmental factors**						
PM_10_(μg/m^3^)	62.03 ± 14.51	30.00	52.00	61.00	71.00	114.00
SO_2_(μg/m^3^)	15.98 ± 7.64	5.00	11.00	14.00	19.00	55.00
NO_2_(μg/m^3^)	30.47 ± 10.01	12.00	23.00	30.00	38.00	66.00
**Demographic**						
PD (people/km^2^)	1451.65 ± 4057.95	56.18	212.29	420.66	688.03	27,823.28
UR (%)	60.01 ± 20.36	29.66	43.24	55.94	70.43	100.00
PEIA (%)	43.36 ± 20.77	0.00	30.78	49.33	58.00	81.85
**Economic factors**						
PGDP(1000 yuan)	57.32 ± 38.56	12.16	29.70	47.46	72.96	258.15
DIUR(1000 yuan)	31.85 ± 6.42	18.11	27.03	31.13	36.66	46.99
DIRR (1000 yuan)	13.71 ± 4.61	4.53	10.75	13.69	16.74	24.87
LINA per 1000 persons	24.03 ± 16.56	2.26	11.32	19.95	30.61	85.31

Abbreviations: *PD* population density, *UR* urbanization rate, *PEIA* proportion of people engaged in agriculture, *PGDP* per capita GDP, *DIUR* disposable income of urban residents, *DIRR* disposable income of rural residents, *LINA* low‑income group in non‑agricultural households

### Spatio-temporal statistical analysis

The Moran'I index of TB incidence from 2014 to 2020 was positive and significant with a mean value of 0.34 and *p*-values less than 0.01, indicating a strong spatial clustering of TB incidence. And the mean value of the lag-1 serial auto-correlation was 0.20, which indicated a degree of correlation in the incidence of TB over time.

**Fig. 3:**
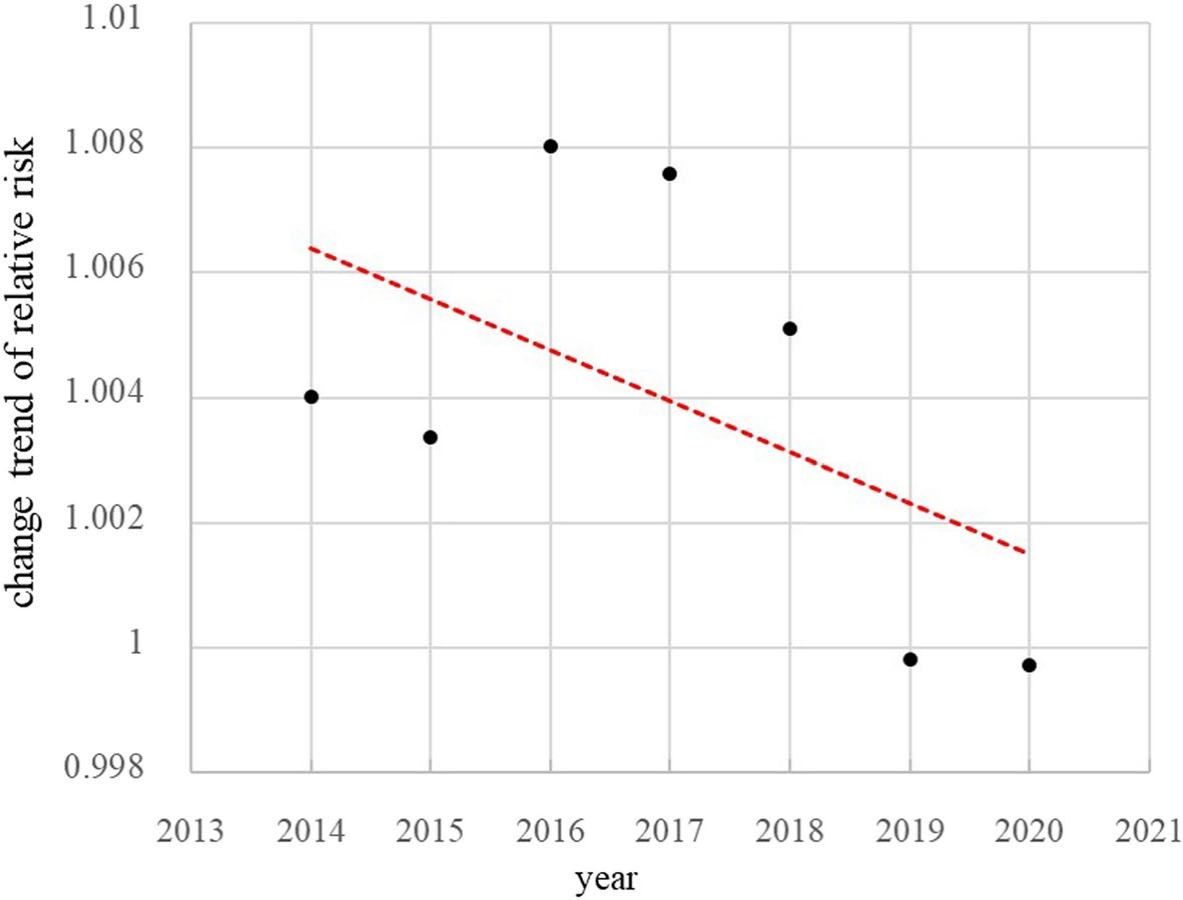
The RR of the temporal effects (exp(g_j_)) from 2014 to 2020

**Fig. 4:**
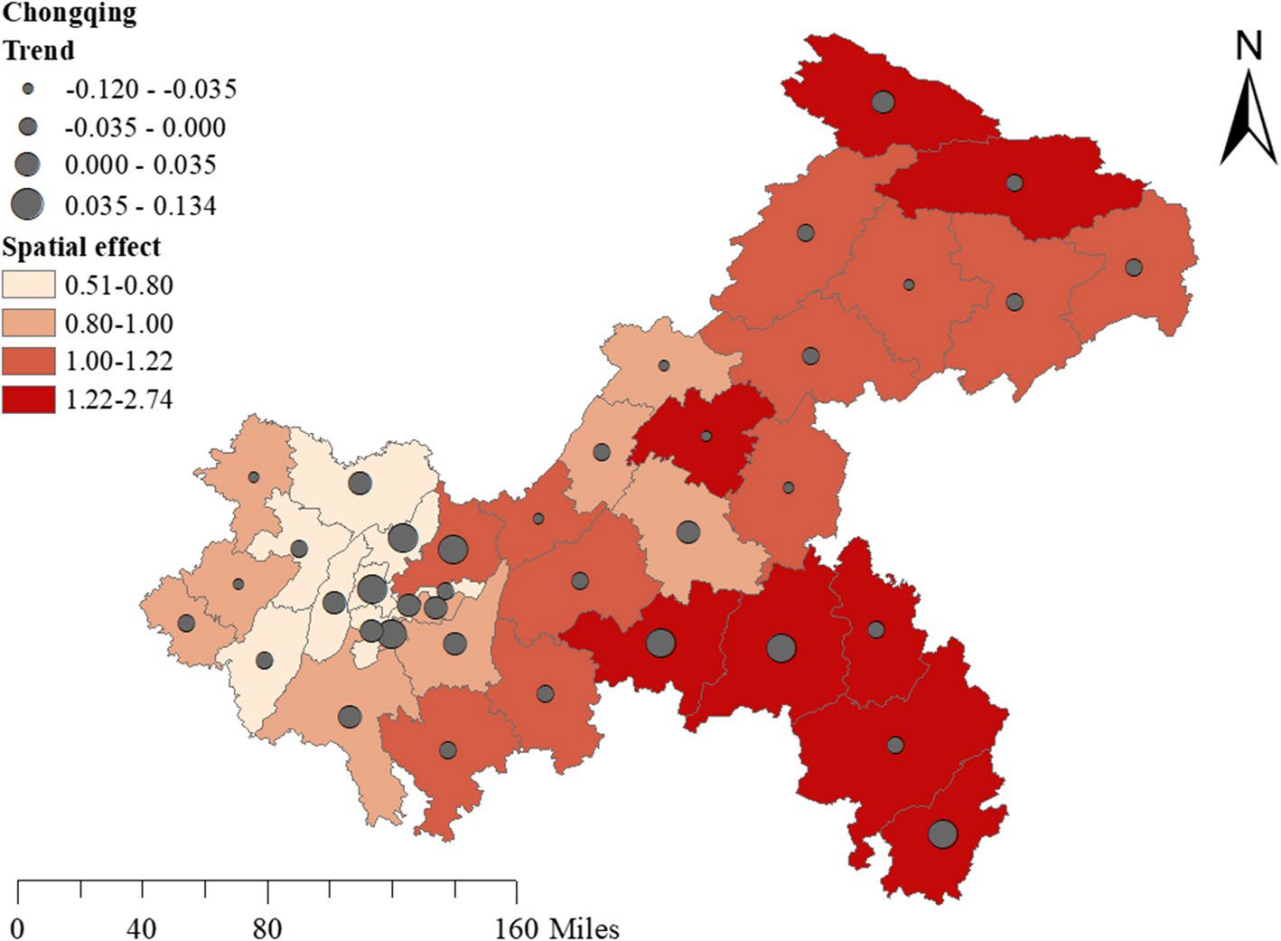
The RR for the spatial effect (exp(u_i_ + v_i_)) and the spatial-temporal interaction effect trend (exp(psi_ij_)) from 2014 to 2020

Table 3 showed that the DIC in model3 had the lowest value among the three Bayesian models. Therefore, the spatio-temporal interaction model was adopted for further analysis. The overall risk of TB incidence showed a downward trend during the study period (Fig. 3). Affected by the spatial effect, the areas with high TB risk incidence were mainly concentrated in the Southeast, which had an upward trend during the study period. At the same time, the areas with low TB risk incidence were in the new urban area, and the risk in the main urban area increased obviously during the period (Fig. 4).

**Table 3:** Deviation Information Criterion (DIC) for the Bayesian model

Bayesian model	Model	DIC
Model1	*θ*_*i*_ = *exp* (*α*_0_ + *u*_*i*_ + *v*_*i*_)	5975.03
Model2	*θ*_*i*_ = *exp* (*α*_0_ + *u*_*i*_ + *v*_*i*_ + *g*_*j*_)	5961.74
Model3	*θ*_*i*_ = *exp* (*α*_0_ + *u*_*i*_ + *v*_*i*_ + *g*_*j*_ + *psi*_*ij*_)	2687.70

**Fig. 5:**
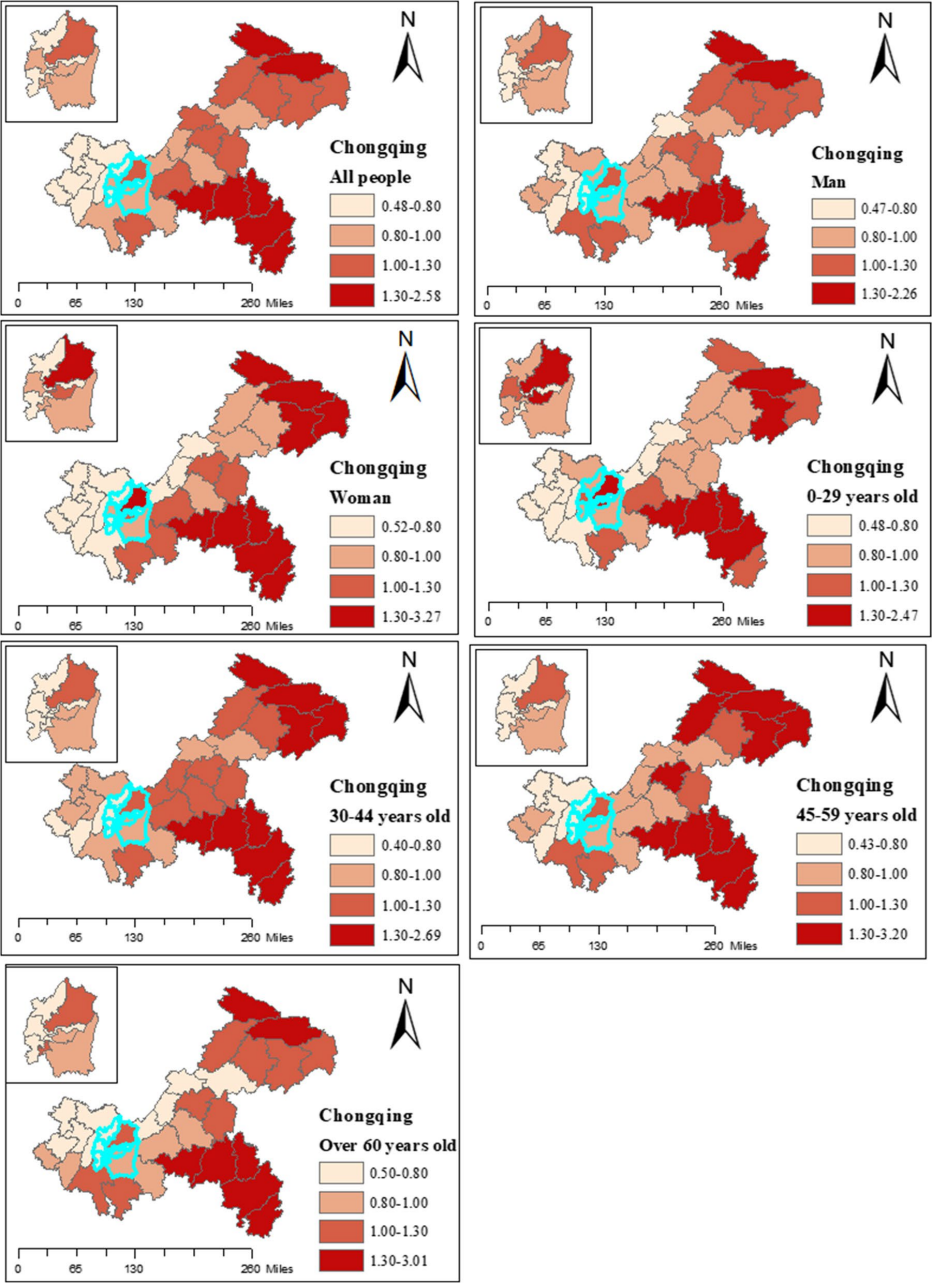
Posterior mean value of TB relative risk (RR) of all and different populations in Chongqing (the small figure showed the main urban area)

**Fig. 6:**
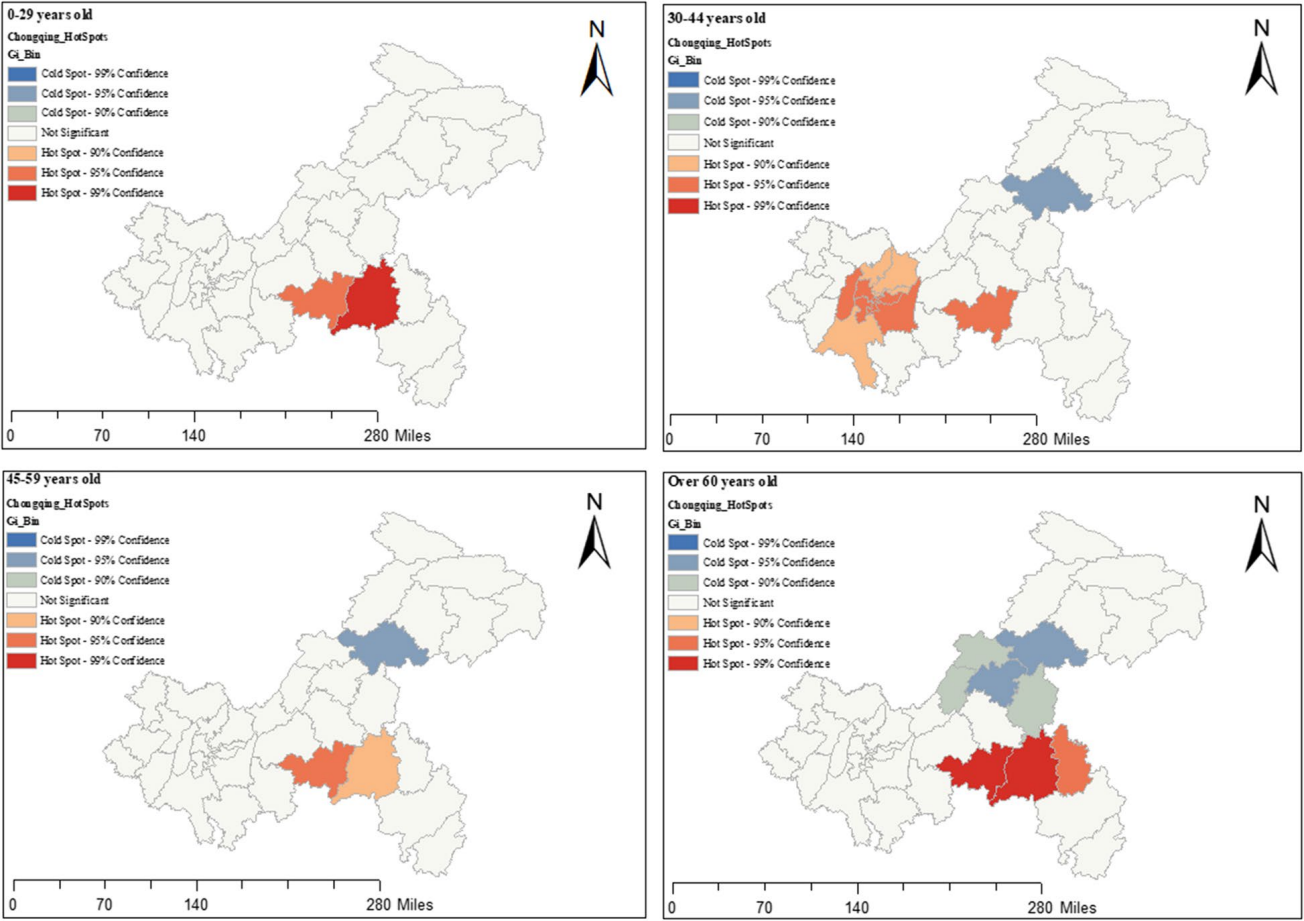
Trend of TB relative risk (RR) with time in different age groups in Chongqing districts(counties)

Figure 5 showed the posterior means of the RR of TB incidence among different populations in different districts (counties) of Chongqing. We could find that women and young people under the age of 30 had a higher RR in main urban area. Figure 6 showed the trend of TB incidence RR with time in different age groups in different districts (counties) of Chongqing. During the study period, the people aged over 60 increased significantly in southeast Chongqing, while the population aged 30 to 44 increased significantly in the main urban area.

### Analysis of influencing factors

We added each environmental and socio-economic variable separately to the Bayesian Spatio-temporal model. Table 4 showed the regression coefficients and 95% confidence intervals (95% CI) estimated by the model. SO_2_ (μg/m^3^), PEIA (%) and LINA (per 1000 persons) were significantly associated with an increase in RR of TB incidence, and UR (%) was significantly associated with a reduction RR of TB incidence (Table 4). Because of the high correlation between the UR (%) and PEIA(r = -0.890), UR (%) was chosen as covariates in the model.

**Table 4:** Univariate analysis of influencing factors of TB

Covariate	DIC	Regression Coefficient	95% credible interval
**Environmental factors**			
PM_10_(μg/m^3^)	2687.60	-0.0003	(-0.0016,0.0099)
SO_2_(μg/m^3^)	2687.07	0.0033	(0.0012,0.0061)
NO_2_(μg/m^3^)	2687.15	-0.0060	(-0.0106,-0.0024)
**Population characteristics**			
PD (people/KM^2^)	2684.30	-0.2025	(-0.2187,-0.1856)
UR (%)	2687.58	-0.2092	(-0.2494,-0.1788)
PEIA (%)	2680.96	0.0125	(0.0091,0.0156)
**Economic factors**			
PGDP(1000 yuan)	2686.91	-0.0404	(-0.1227,0.0442)
DIUR(1000 yuan)	2686.93	-0.0025	(-0.0083,0.0035)
DIRR (1000 yuan)	2688.08	-0.0041	(-0.0156,0.0047)
LINA (per 1000 persons)	2687.49	0.0661	(0.0319,0.1106)

Abbreviations: *PD* population density, *UR* urbanization rate, *PEIA* proportion of people engaged in agriculture, *PGDP* per capita GDP, *DIUR* disposable income of urban residents, *DIRR* disposable income of rural residents, *LINA* low‑income group in non‑agricultural households

Based on the results of the Bayesian spatio-temporal model with the final addition of SO_2_, UR and LINA, it was found that the increase in SO_2_ was associated with an increased RR of TB incidence in the entire population. 1 μg/m^3^ increase in SO_2_ was associated with 0.35% increase in RR of TB incidence (95% CI: 0.08–0.61%). The increase in UR was associated with reduced RR of TB incidence in the entire population, with each 1% increase in UR associated with 0.15% (95% CI: 0.11–0.17%) reduction in the RR of TB incidence. However, the effect of this variable was reversed for the female population and those under 30 years of age, with each 1% increase in UR associated with 0.18% (95% CI: 0.16–0.21%) and 0.37% (95% CI: 0.34–0.45%) increase in the RR of TB incidence, respectively. The increase in the LINA was associated with increased RR of TB incidence, with each 1% increase in the LINA associated with 0.07% increase in the RR of TB incidence(95% CI: 0.05–0.10%). And the variable had a greater impact on TB infection in the female population and those aged 60 years or older, with each 1% increase in the LINA associated with 0.16% (95% CI: 0.10–0.20%) and 0.17% (95% CI: 0.15–0.21%) increase in the RR of TB incidence, respectively (Table 5).

**Table 5:** Multivariate analysis of influencing factors of TB

Covariate	Regression Coefficient	95% credible interval
**The whole crowd**		
SO_2_(μg/m^3^)	0.0035	(0.0008,0.0061)
UR (%)	-0.1521	(-0.1721,-0.1097)
LINA (per 1000 persons)	0.0703	(0.0528,0.0958)
**Male**		
SO_2_(μg/m^3^)	0.0019	(0.0002,0.0036)
UR (%)	-0.1651	(-0.1835,-0.1505)
LINA (per 1000 persons)	0.0511	(0.0383,0.0653)
**Female**		
SO_2_(μg/m^3^)	0.0018	(-0.0014,0.0071)
UR (%)	0.1786	(0.1582,0.2102)
LINA (per 1000 persons)	0.1555	(0.0968,0.1958)
**People aged 0–29**		
SO_2_(μg/m^3^)	0.0046	(0.0018,0.0080)
UR (%)	0.3653	(0.3379,0.4493)
LINA (per 1000 persons)	0.0962	(0.0171,0.1505)
**People aged 30–44**		
SO_2_(μg/m^3^)	0.0043	(-0.0017,0.0088)
UR (%)	-0.5061	(-0.5258,-0.4832)
LINA (per 1000 persons)	0.1261	(0.0801,0.1604)
**People aged 45–59**		
SO_2_(μg/m^3^)	-0.0017	(-0.0061,0.0050)
UR (%)	-0.3583	(-0.4074,-0.3164)
LINA (per 1000 persons)	0.0657	(0.0153,0.1221)
**60-Crowd**		
SO_2_(μg/m^3^)	0.0030	(-0.0011,0.0068)
UR (%)	-0.2108	(-0.2626,-0.1818)
LINA (per 1000 persons)	0.1744	(0.1476,0.2139)

Abbreviations: *UR* urbanization rate, *LINA* low‑income group in nonagriculturalhouseholds

## Discussion

In this study, a Bayesian spatio-temporal model was developed to analyze the risk of TB incidence in Chongqing from 2014 to 2020, to explore the spatio-temporal incidence patterns of TB in different populations, and to identify potential correlates of TB incidence.

Chongqing reported a total of 170,934 TB cases from 2014 to 2020, with the morbidity per 100,000 people decreasing from 82.81 in 2014 to 70.19 in 2020, but higher than the national average in all years [4]. The elderly population aged over 60 years old was a key population with a high incidence of TB, and the risk of TB incidence was on the rise in the elderly population in southeast Chongqing. This might be related to the lower literacy level and poor health literacy of the elderly, especially in economically backward areas such as southeastern Chongqing, where with the exodus of young and middle-aged groups, aging and empty-nesting were becoming more serious, and the local elderly left behind had even less health awareness and lacked of knowledge about TB prevention and treatment [29]. In this regard, it is recommended that primary healthcare institutions should popularize knowledge about TB disease among the elderly, provide free consultations to empty nesters regularly, and implement early detection and treatment of TB in this group. In addition, there was an increasing trend in the risk of TB incidence in main urban areas among those aged 30 to 44 years. This might be due to the dramatic increase in youth mobility in main urban areas in recent years [29], but the corresponding housing conditions and medical resources were relatively poor. Therefore, it is recommended that the government improves the living environment of the urban population, especially the mobile population, and improves the primary health service and medical security system to reduce the burden of TB disease in this population.

The study found a positive effect of SO_2_ on the development of TB, which was consistent with the study in Hubei, China, and this might be related to the fact that SO_2_ caused death of alveolar macrophages and reduced the ability of the immune system to clear Mycobacterium TB [11]. In China, SO_2_ emissions from coal combustion were a major problem, especially in winter when heating increases SO_2_ emissions [30]. In Chongqing, due to the mountainous terrain, the low wind speed in winter made it difficult for air pollutants to escape, which aggravates the impact of SO_2_ emissions on the development of TB. This study also found that there was a clear seasonal trend in the number of TB cases, mainly concentrated in January–March each year, which might be related to the increased SO_2_ concentration in winter. Therefore, in order to control air pollution at the source and reduce the possibility of TB incidence, it is recommended to vigorously promote “coal to gas” or “coal to electricity” in all districts and counties, especially in rural areas, or to use sulfur-free coal to reduce SO_2_ emissions [30].

High UR was also found in the study to have a significant negative effect on the increased risk of TB incidence in the whole population. This might be attributed to areas with high UR had more knowledge of TB control, good health awareness, and better health care for their residents. However, further subpopulation analysis found a significant positive effect of high UR on TB incidence in the female population. Analysis of its possible causes: First, women in areas with high UR have higher employment rates [31], and employment might increase their chances of contracting Mycobacterium TB, especially in main urban areas with high mobile populations. Our findings also showed that women had a higher risk of TB incidence in main urban areas. Secondly, environmental pollution, traffic congestion and deteriorating living conditions brought about by urbanization may also accelerate the infection and spread of Mycobacterium TB. In addition, high UR increased the risk of morbidity in people aged 0–29 years. Students accounted for a large portion of the population, and the concentration of classes and housing on campus makes it extremely easy for the spread of Mycobacterium TB and the spread of infection. At the same time, the increased work pressure and work style changes brought by urbanization might bring more physical and psychological stress to the youth population [32], and a chronic state of physical suboptimal health, which may increase the risk of TB. In this regard, the government should emphasize the coordination between the speed of development and the quality of people’s living standards, optimize the spatial layout of cities and towns, improve the living and working environment of residents, and realize healthy urbanization.

In the Global TB Report 2021, it was suggested that poverty was a determinant of the number of people infected with TB disease [2, 33]. Similar results were obtained in the present study, where we found a significant positive effect of the increase in LINA on the increase in TB risk. Some studies have shown that people in poor areas had lower access to health services and were more likely to have poor nutrition and poor behavioral habits such as smoking and alcohol consumption [34], which increased the risk of TB infection. Therefore, it is suggested that the government should increase the medical protection and assistance for people in poor areas, and strengthen the improvement of their diet and nutrition and the guidance of exercise, so as to promote the development of healthy habits and enhance the physical quality of such people. Subgroup analysis further revealed that the female population and the older age group over 60 were more affected by poverty. In resource-limited conditions, elderly and female populations may be more vulnerable, especially due to folk customs and patriarchal beliefs, and they may have poorer health status and lower access to health services [35, 36], thus increasing the risk of TB incidence.

We had several strengths in our research. First, the Bayesian spatio-temporal model used in this paper took the spatio-temporal structure as a priori, considered the influence of adjacent temporal and spatial structures, realized the simultaneous analysis of temporal, spatial, and spatio-temporal interactions and potential influencing factors of TB, and considered more uncertainties, so that the risk of TB incidence could be estimated more accurately, and the pattern of spatio-temporal variation of its incidence and its influencing factors could be analyzed. Second, differences in the spatial and temporal distribution of TB risk in different populations were found in the results of this study. The female population and the young people aged 0–29 years were at higher risk of TB in the main urban area, and there was an increasing risk for people aged 30–44 years in the main urban area and for people over 60 years old in the southeast of Chongqing. These results provided additional evidence and insight to specifically analyze the distribution patterns of TB incidence in different populations. Third, the results of this paper confirmed the effects of air pollution factors and socio-economic factors on the incidence of TB, and further explored the differences in the effects of these factors on different populations. The increase in UR had a significant positive effect on the increased risk of TB in the female population and the youth population, while it had a negative effect on the rest of the population. The LINA per 1000 persons had a greater impact on the risk of TB in the female population and in the population aged 60 years or older than in other populations.

There were also some limitations in our analysis. First, although several environmental and socio-economic variables were included in our study, there were a number of potential influences on TB incidence that were not included, such as smoking rates, alcohol consumption rates, etc. Second, the TB cases reported in this study were from the TB surveillance system, which might have been influenced by the lack of reporting and underdiagnosis in some poor counties, leading to an underestimation of TB incidence. Finally, the analysis level of this study was divided according to districts (counties). We can consider using smaller geographical units, such as streets and towns to explore the districts with a high risk of TB incidence like Southeast Chongqing and Northeast Chongqing in a future study.

## Conclusion

This study showed that high-risk areas for TB were concentrated in the southeastern and northeastern regions of Chongqing, and the elderly population was a key population for TB incidence. There were spatial and temporal differences in TB incidence among populations with different characteristics, with women and young people having a higher risk of incidence in main urban area and those aged over 60 years of age having an increasing risk of incidence in southeastern Chongqing, and this difference may be due to environmental, demographic and economic factors. Local governments should focus on areas and populations at high risk of TB incidence and develop targeted prevention interventions based on the characteristics of different populations.

## Data Availability

In order to protect the privacy of TB patients, we will not share the original copies of information from the database. We would like to share the statistical results of this study. If anyone needs these data, please contact the corresponding author for a soft copy.

## Abbreviations

TB: Tuberculosis
RR: Relative risk
CDC: Center of disease control and prevention
PM_10_: Particles with an aerodynamic diameter less than or equal to 10 μm
SO_2_: Sulfur dioxide
NO_2_: Nitrogen dioxide
PD: Population density
UR: Urbanization rate
PEIA: Proportion of people engaged in agriculture
PGDP: Per capita GDP
DIUR: Disposable income of urban residents
DIRR: Disposable income of rural residents
LINA: Low-income group in non-agricultural households
SD: Standard deviation
CAR: Conditional auto-regression
DIC: Deviance information criterion
P25: 25% quartile
P75: 75% quartile
